# No Association Between *AGT* Gene Polymorphisms with Hypertension in a South African Population

**DOI:** 10.2147/DMSO.S452272

**Published:** 2024-04-29

**Authors:** Jyoti Rajan Sharma, Hannah Fokkens, Ria Laubscher, Teke Ruffin Apalata, Sibusiso Cyprian Nomatshila, Samuel Yao Alomatu, Hans Strijdom, Rabia Johnson

**Affiliations:** 1 Biomedical Research and Innovation Platform, South African Medical Research Council, Cape Town, South Africa; 2 Centre for Cardiometabolic Research in Africa, Division of Medical Physiology, Faculty of Medicine and Health Sciences, Stellenbosch University, Cape Town, South Africa; 3 Biostatistics Research Unit, South African Medical Research Council, Cape Town, South Africa; 4 Department of Laboratory-Medicine and Pathology, Faculty of Health Sciences, Walter Sisulu University and National Health Laboratory Services, Mthatha, South Africa; 5 Department of Public Health, Faculty of Medicine and Health Sciences, Walter Sisulu University, Mthatha, South Africa; 6 Department of Internal Medicine, Nelson Mandela Central Hospital and Walter Sisulu University, Mthatha, South Africa

**Keywords:** hypertension, gene, ACE, AGT, polymorphism

## Abstract

**Purpose:**

Hypertension is a leading cause of cardiovascular-related morbidity and mortality worldwide, with a prevalence increasing at an alarming rate in both middle- and low-income countries. Various environmental and genetic factors have been attributed to play a significant role in the increasing prevalence of hypertension. Single nucleotide polymorphisms (SNPs) in the angiotensinogen (*AGT*) gene are reported to have a significant association with hypertension; however, there are limited studies done on South African populations. Therefore, this case–control study aimed to investigate the association between *AGT* SNPs (rs2004776, rs3789678, rs5051 and rs7079) with hypertension in a study population of isiXhosa-speaking participants from the Eastern Cape Province in South Africa.

**Materials and Methods:**

The SNPs were genotyped in 250 hypertensive cases and 237 normotensive controls, using TaqMan genotyping assays.

**Results:**

For the SNP rs2004776, the frequency of CC genotype (18.4%) and C allele (44%) in hypertensive cases showed no significant differences (p = 0.52, χ2 = 1.32), when compared to the normotensive control group (CC: 19.8% and C allele: 43%). Similar results were obtained for the genotypic and allelic frequencies between hypertensive cases and normotensive controls for rs3789678 (p = 0.88, χ2=0.26) and rs5051 (p = 0.57, χ2=1.12), and rs7079 (p = 0.33, χ2=2.23). These findings demonstrate that there were no significant associations between the SNPs rs2004776, rs3789678, rs7079, rs5051 with hypertension in our study population.

**Conclusion:**

These findings suggest that *AGT* gene polymorphisms are not associated with the development of hypertension in the studied population. The present study represents the first genetic report to investigate the *AGT* gene polymorphisms with hypertension in an isiXhosa-speaking South African population.

## Introduction

Cardiovascular diseases (CVD) are the leading cause of deaths globally, accounting for 19.8 million deaths worldwide in 2022.^1–3^ Hypertension drives the global burden of CVD and is a leading cause of cardiovascular-related mortality worldwide, with 1.39 billion affected adults and 10.4 million deaths globally.^4–6^ It has emerged as a major public health issue in sub-Saharan Africa, contributing to the region’s rising number of early deaths. In South Africa, hypertension has an estimated prevalence of 27–58% and is referred as a “silent killer”, with 46% of adults being oblivious of their hypertension status.^7–11^ The prevalence of hypertension in the rural communities is even higher. According to Sharma et al,^12^ the prevalence of hypertension is 71% in the rural community of Mthatha town in Eastern Cape province, South Africa. Previously, other studies have also highlighted the serious concern of escalating prevalence of hypertension in rural South Africa.^13–15^ Despite having an advanced understanding of the pathogenesis and the management of hypertension, there is still a progressive rise in its prevalence.^16^

Various factors, including environmental and genetic factors, have been attributed to play a significant role in the increasing prevalence of hypertension.^12^,^17–19^ Although the exact role of the genetic factors in the regulation of hypertension is still unknown, the genes encoding the main components of the renin-angiotensin-aldosterone system (RAAS) are deemed the most biologically plausible genes.^20^,^21^ The RAAS hormonal system is involved in long-term blood pressure regulation^22^,^23^ and in the aetiology of hypertension by controlling sodium and water retention, oxidative stress, fibrosis, and inflammation, as well as systolic blood pressure.^24^ RAAS regulates blood pressure via a cascade of reactions involving the activation of various key proteins.^25^

Proteins such as angiotensinogen (gene: *AGT*, protein: AGT), angiotensin-converting enzyme (gene: *ACE*, protein: ACE), renin, angiotensin I, angiotensin II, angiotensin receptor type I, and angiotensin receptor type II are significant components of RAAS. AGT is a source of all the angiotensin peptides, and renin cleaves AGT into angiotensin.^26^,^27^ The ACE then cleaves angiotensin I into angiotensin II, which causes vasoconstriction and inhibits bradykinin-modulated vasodilation, resulting in elevated blood pressure.^28^

Polymorphisms in the *AGT* gene are reported to have significant associations with hypertension.^17^,^29^,^30^ The *AGT* (1q42-q43) gene encode for the AGT protein.^31^ SNPs within the *AGT* gene, such as rs3789678 (Guanine (G)→ Adenine (A) at position 230713736), and rs2004776 (Thymine (T) → Cytosine (C) at position 230712956), rs5051 (Cytosine (C) → Adenine (A) at position 230714126), and rs7079 (Guanine (G) → Thymine (C) at position 230702585), have been investigated for their causal role in hypertension.^32^

In a meta-analysis,^21^ it was reported that only a limited number of studies have evaluated the involvement of *AGT* gene with the development of hypertension in African populations. To date, only one study^17^ reported a significant association between rs2004776 (*AGT* gene) SNP with hypertension in a Ugandan population. However, other studies performed in African populations exhibited no association between *AGT* gene polymorphisms and risk of developing hypertension.^20^,^33–35^ Furthermore, considering the reduced efficacy of angiotensin receptor blockers (ARBs) in individuals of African ancestry,^36^,^37^ a better understanding of genetic differences is imperative to evaluate disease risk and differential drug response of patients of African ancestry to antihypertensive medications. It has also been suggested that indigenous African populations have higher level of genetic diversity as compared to the other populations of the world.^38^ Therefore, the genomic studies of ethnically and genetically diverse populations are critical to understand the differential disease risk in humans.^39^ However, there is a paucity of data pertaining to the association of SNPs and the pathogenesis and treatment of hypertension in the rural communities of South Africa.^21^,^40^

Therefore, the aim of this study was to investigate the putative role of *AGT* gene in hypertension status. This was achieved by genotyping *AGT* SNPs (rs2004776, rs3789678, rs5051 and rs7079) in an isiXhosa-speaking South African population from the Eastern Cape, South Africa. We hypothesize that these polymorphisms are associated with the increased risk of developing hypertension.

## Materials and Methods

### Study Design

The present case–control study included isiXhosa speaking participants from four rural districts (OR Tambo, Alfred Nzo, Chris Hani, Joe Gqabi), and five subdistricts, involving fourteen community health centres in Mthatha in the Eastern Cape Province of South Africa. As per the provincial profile of Eastern Cape,^41^ the OR Tambo district has the highest population density of 112 people per km², followed by Alfred Nzo, Chris Hani and lastly Joe Gqabi with a density of 13 people per km². Approximately half of the community health facilities involved in this study were situated in rural areas, defined by the Rural Development Framework as sparsely populated areas in which people depend on natural resources or farming, including villages and small towns dispersed throughout these areas”. The percentage of persons aged 0–34 years varied from 36–40%; however, OR Tambo and Alfred Nazo had 13% of the population in 35–64 age group, while this percentage was higher in both Chris Hani and Joe Gqabi districts (approximately 15%). The proportion of population over 65 years was the highest in Chris Hani district (8.6%) followed by Alfred Nazo (7.9%), Joe Gqabi (7.5%), and OR Tambo (6.5%). All the district municipalities are inhabited mainly by the black African population. Sex ratio depicted that at young ages (0–19 years), there were more males as compared to females; however, the age group >20 years reported more females.

The highest percentage of residents in these districts reported that they speak isiXhosa language and are affiliated to Christianity. In OR Tambo and Chris Hani and Alfred Nazo district over 60% aged 18 years were never married. However, Joe Gqabi had the lowest percentage of never married persons.

### Eligibility Criteria

This study included only adult participants (over 18 years of age or more) and excluded pregnant women and those who were reluctant to participate.

### Ethical Clearance

The study was conducted following the Declaration of Helsinki^42^ and approved by the Institutional Review Board (or Ethics Committee) of SAMRC (SAMRC: EC028-8/2020), Water Sisulu University (073/15) and Stellenbosch University Human Research ethics committee (S22/05/095). The essential information about the participants’ rights, research purpose, and research process was provided to the participants in two different languages (English and isiXhosa) before obtaining the informed consent.

### Data Collection

Data were collected via the World Health Organization (WHO) STEPwise amended questionnaire and deposited into the Research Electronic Data Capture system (REDCap™). Data collected included sociodemographic factors (age, gender, ancestry) and anthropometric measurements (waist circumference, hip circumference, height, weight) and blood pressure measurements (systolic blood pressure, diastolic blood pressure). The most important determinants of hypertension from other studies^43^ and significant determinants identified in the previous study on the same population were included.^12^

### BMI and Obesity Profiles

Anthropometric measurements such as body weight and height were used to calculate body mass index (BMI) and waist-to-hip ratio (WHR). A conventional beam balance was used to weigh barefoot participants in light clothing to the nearest 0.1 kg. On a mounted stadiometer, height was measured to the nearest 0.1 cm, and BMI was determined by using the following formula: weight/(height)². According to the WHO standards, participants were categorized as normal, overweight, and obese.^44^,^45^ Participants were considered overweight if BMI was 25–29.9 kg/m^2^ and were categorized as obese if their BMI was ≥30.0 kg/m^2^. Participants with BMI ≤ 25 were either classified as normal or underweight. The waist circumference measurement was taken approximately halfway between the lower edge of the last palpable rib and the top of the iliac crest.^46^ The measurement of the hip circumference was obtained around the broadest part of the buttocks. The waist to hip ratio was calculated using the following formula: waist circumference/hip circumference.

### Diagnosis of Type 2 Diabetes Mellitus (T2D)

A blood sample was taken from each participant after an overnight fast. The diagnosis of T2D was confirmed according to the criteria of the American Diabetic Association, 2022.^47^,^48^ Participants were categorized as normal (fasting blood sugar level < 5.6 mmol/L), prediabetic (fasting blood sugar level between 5.6 - to 6.9 mmol/L), and diabetic (fasting blood sugar level >7 mmol/L).

### Measurement and Definition of Blood Pressure

Before blood pressure measurement, participants were asked to sit at rest for approximately five minutes. Blood pressure was measured using a slight touch ST-401 blood pressure monitor based on standard operating procedures. Diagnosis of hypertension status was defined as per the South African Hypertension Society guidelines (Seedat, Rayner and Veriava, 2014). Participants were categorised as follows: (1) Normal blood pressure (systolic blood pressure (SBP): <120 mm Hg and diastolic blood pressure (DBP) < 80mm Hg (2) optimal blood pressure (SBP: 120–129 mm Hg and DBP < 80 mm Hg), (3) High Normal (SBP: 130–139 mm Hg and DBP: 80–89 mm Hg), and (4) Hypertension (HTN) grade 1 SBP ≥ 140 mm Hg or DBP ≥ 90 mm Hg. Based on these guidelines, the participants in the current study were categorized as normotensive (SBP ≤ 120mm Hg; DBP < 80mmHg) or hypertensive based on the values of blood pressure (SBP ≥ 140-159mm Hg or DBP: ≥90-99mm Hg) or if they are already on prescribed antihypertensive medication. The diagnosis of hypertension was confirmed after evaluating the patients’ medical history, symptoms, and prescribed medication for three months.

### Sampling Procedure

We enrolled a total of 487 participants (237 normotensive controls and 250 hypertensive cases). With written and informed consent, venous blood (5 mL) was collected in EDTA tubes (anticoagulant). The samples were stored at −80 degrees Celsius until further analysis. A blood sample was also taken after an overnight fast to measure the glucose levels.

### DNA Extraction and Genotyping

Genomic DNA was extracted from 1mL of whole blood sample, using the QIAamp Blood Midi Kit (Qiagen, Hilden, Germany) according to the manufacturer’s instructions. The extracted DNA was quantified on the NanoDrop Spectrophotometer (Nanodrop Technologies). The selection of the variants (rs2004776, rs3789678, rs5051, rs7079) of *AGT* gene was based on the previous literature and with minor allele frequency (MAF) >1% in the population upon referring to the public databases. There is paucity of data on the association of these SNPs in African population. The genotypes were determined using quantitative Real-Time PCR (qRT-PCR) with TaqMan genotyping assays (Applied Biosystems, Massachusetts, United States). Four SNPs located in *AGT* (rs2004776, rs3789678, rs5051, rs7079) were included in the study, and primers were designed as shown in Table 1. The File Builder tool was then used to order primers from Applied Biosystems and synthesized according to the specifications. The TaqMan custom genotyping assay probes (x40) were assigned the labels VIC and FAM to the sequences below (Table 1).

All samples were diluted to a final concentration of 10 ng/µL and aliquoted individually (4.75 µL) in each well of a 96 well plate. A PCR master mix was prepared by adding 5 µL of TaqPath ProAmp Master Mix and 0.25 µL of 40X TaqMan SNP Genotyping Assay in a total volume of 10 µL, according to the manufacturer’s instructions (Applied Biosystems). A volume of 5.25 µL of the master mix was added to each well, yielding a final volume of 10 µL (4.75 µL sample + 5.25 µL master mix) in each well. Negative control of the same volume was also added with each PCR run. The Quant studio 5 (Applied Biosystems, Massachusetts, United States) was used for the PCR amplification. PCR parameters were as follows: pre-read for 30s at 60°C (hold); initial denaturation for 5 min at 95°C (hold); denaturation for 5s at 95°C for 40 cycles; annealing/extension for 30s at 60°C for 40 cycles; post-read for 30s at 60°C (hold). All samples were genotyped in duplicate. The QuantStudio^TM^ 7 design and analysis software was for base calling and visualization of the genotyping data (Supplementary Figure S1).

**Table 1:** Details of Single Nucleotide Polymorphisms Assays

Gene	Variant	Nucleotide Position	SNP Sequence	Global MAF	AFR MAF
AGT	rs2004776	Chromosome 1	GGCTGAATGCTAAAGGTGAAGATGA [C/T] GGCTCATGCTCCTGTGGTGCCTGCC	C=0.59	C=0.51
		230,712,956		T=0.41	T=0.49
	rs3789678	Chromosome 1	CCTTCCCTCCAGCCCCAATTCCTG [C/T] ACAAGCCCTGCTATTCCTCCTGATG	C=0.82	C=0.75
		230,713,736		T=0.18	T=0.25
	rs5051	Chromosome 1	CCAGAACAACGGCAGCTTCTTCCCC [C/T] GGCCGGGTCACGATGCCCTATTTAT	T=0.71	T=0.92
				C=0.29	C=0.08
		230,714,126			
	rs7079	Chromosome 1	ACACAAGGGAGAAATAACCAGCTAT [G/T] GTTCCGCATTCAAACAGAAATTCAG	G=0.81	G=0.93
		230,702,585		T=0.19	T=0.07

**Notes**: Bold letters indicate the SNP of interest in each sequence. Global MAF: minimum allele frequency at the global level. AFR MAF: minimum allele frequency in Africa.

### Statistical Analysis

Clinical and anthropometric parameters were presented as mean ± standard deviation (SD) if normally distributed and as median (Q1–Q3) if skewed. Shapiro-Francia W’ test was used to assess normality. In the case of normally distributed parameters, Student’s *t*-test was used, and for skewed parameters, median regression (QREG in STATA) was used to assess group differences (hypertensive vs control group), for the pooled sample and within gender. Allelic and genotypic frequencies were compared. The conformity of genotype distributions was evaluated using Hardy-Weinberg equilibrium. The association between hypertension and genotype was tested using the chi-squared test and odds ratio with 95% confidence intervals was calculated to evaluate allelic association with the disease status. Logistic regression models enabled us to adjust for age, gender, and waist circumference (WC). This was done for the various inheritance models (dominant, recessive, and codominant), and the multivariate model. Statistical analysis was performed using the STATA16 (Software for Statistics and Data Science, package SE).

## Results

The demographic, clinical, and anthropometric parameters of 487 participants (237 normotensive controls and 250 hypertensive cases) are presented in Table 2. The hypertensive cohorts had a higher age mean of 55 years as compared to normotensive controls (mean = 41.6 years), with the differences being significant in the total pooled (p < 0.001) groups. All parameters were significantly higher (p < 0.05) in hypertensive cases as compared to normotensive controls in the total pooled sample. Furthermore, it was demonstrated that gender is not a statistically significant predictor of developing hypertension (p = 0.320).

**Table 2:** Comparison of Various Parameters (Mean ± SD) in Total Pooled, Male, and Female Hypertensive Cases and Normotensive Controls

Variable	Hypertensive Cases N=250	Normotensive Controls N=237	Total N=487	p-value
**Gender**				
**Female (N)**	205 (83.3%)	186 (79.8%)	391 (81.6%)	0.320
**Male (N)**	41 (16.7%)	47 (20.2%)	88 (18.4%)	
**Age (years)**	55.13 (13.08)	41.64 (12.74)	48.54 (14.56)	<0.001*
**BMI (kg/m^2^)**	32.35 (6.79)	27.98 (6.65)	30.11 (7.06)	<0.001*
**WC (cm)**	102.03 (13.91)	91.06 (13.66)	96.67 (14.83)	<0.001*
**HC (cm)**	113.67 (13.91)	107.78 (13.49)	110.78 (14.00)	<0.001*
**WHR**	0.90 (0.09)	0.85 (0.09)	0.87 (0.09)	<0.001*
**Blood Glucose (mmol/L)**	6.58 (3.13)	5.76 (2.06)	6.17 (2.68)	<0.001*
**SBP (mm Hg)**	142.88 (21.22)	117.64 (10.78)	130.50 (21.10)	<0.001*
**DBP (mm Hg)**	89.64 (11.80)	75.69 (7.18)	82.80 (12.03)	<0.001*

**Note**: *p < 0.05 statistically significant.

**Abbreviations**: BMI, Body Mass Index; SBP, Systolic Blood Pressure; DBP, Diastolic Blood Pressure; WHR, Waist to Hip Ratio WC – waist circumference; HC, Hip circumference.

### Genotyping and Allele Frequencies

A univariate analysis was used to identify SNP association with the disease status, and the data were adjusted for age, BMI and waist circumference. The genotype distribution conformity was assessed using the Hardy-Weinberg equilibrium, and it was observed that genotypes in both cases and controls were in equilibrium. Genotypic and allelic frequencies in hypertensive subjects and normotensive controls for the studied SNPs are given in Table 3. For the SNP rs2004776, no significant differences were observed for genotypic and allelic frequencies between hypertensive cases and normotensive controls (p = 0.52, χ2 = 1.32). Similarly, the genotypic and allelic frequencies between hypertensive cases and normotensive controls were not significantly different for rs3789678 (p = 0.88, χ2 = 0.26) and rs5051 (p = 0.57, χ2 = 1.12), and rs7079 (p = 0.33, χ2 = 2.23). Univariate analysis demonstrated that the studied SNPs (rs2004776, rs3789678, rs5051, and rs7079) were not associated with the risk of developing hypertension.

**Table 3:** Genotypic Frequency and Allelic Frequency Distribution for *AGT* gene Polymorphisms in Hypertensive Cases and Normotensive Controls

Genotype/allele Frequency	Hypertensive Cases N=250	Normotensive Controls N=237
**rs2004776**		
**CC (homozygous wild type)**	46 (18.4%)	47 (19.8%)
**CT (heterozygous)**	118 (47.2%)	120 (50.6%)
**TT(homozygous mutant type)**	86 (34.4%)	70 (29.5%)
**C**	44%	43%
**T**	53%	60%
**Univariate analysis**	OR=1.14 (0.79–1.63) | p=0.52, χ2=1.32	
**rs3789678**		
**CC** (**homozygous wild type)**	101 (40.4%)	93 (39.2%)
**CT (heterozygous)**	113 (45.2%)	106 (44.7%)
**TT (homozygous mutant type)**	36 (14.4%)	38 (16.0%)
**C**	60%	65%
**T**	37%	38%
**Univariate analysis**	OR=0.94 (0.65–1.36) | p=0.88, χ2=0.26	
**rs5051**		
**TT (homozygous wild type)**	218 (87.2%)	204 (86.1%)
**CT (heterozygous)**	32 (12.8%)	32 (13.5%)
**CC (homozygous mutant type)**	0 (0.0%)	1 (0.4%)
**T**	90%	90%
**C**	7%	7%
**Univariate analysis**	OR=1.13 (0.56–2.29) | p=0.57, χ2=1.12	
**rs7079**		
**GG (homozygous wild type)**	227 (90.8%)	209 (88.2%)
**GA (heterozygous)**	7 (2.8%)	13 (5.5%)
**AA (homozygous mutant type)**	16 (6.4%)	15 (6.3%)
**G**	88%	95%
**A**	9%	8%
**Univariate analysis**	OR=0.85 (0.45–1.61) | p=0.33, χ2=2.23	

**Abbreviation**: OR, Odds Ratio for allele frequency and χ^2^ for genotype frequency.

### Inheritance Models and Univariate Analysis

Various hypothetical inheritance models were used to analyse the association of each SNP with the disease status (Table 4). For SNP rs2004776, under the dominant (p = 0.609), recessive (p = 0.865), and codominant (p = 0.577) inheritance models, the T allele did not confer any risk of developing hypertension. The analysis of rs3789678 polymorphism also demonstrated no significant association of the risk allele with disease status, under dominant (p = 0.356), recessive (p = 0.674), and codominant (p = 0.545) inheritance models. Similar results were obtained for the SNP rs7079 under the dominant (p = 0.912), recessive (p = 0.953), and codominant model (p = 0.805). For rs5051, the number of subjects with genotype CC was very low; therefore, this SNP was not included in the further analysis.

**Table 4:** Association Between AGT Polymorphisms and Hypertension Status in Different Inheritance Models

	Unadjusted OR (95% CI)	Unadjusted p	aOR (95% CI)	Adjusted p
**rs2004776**				
CC vs CT+TT Dominant model	0.91 (0.58–1.43)	0.688	1.16 (0.66–2.04)	0.609
TT vs CT+CC Recessive model	1.25 (0.85–1.83)	0.251	1.04 (0.65–1.68)	0.865
CT vs CC+TT Codominant model	0.87 (0.61–1.24)	0.449	0.88 (0.57−1.37)	0.577
**rs3789678**				
CC vs CT+TT Dominant model	1.05 (0.73–1.50)	0.794	1.24 (0.79–1.94)	0.356
TT vs CT+CC Recessive model	0.88 (0.54–1.45)	0.616	0.88 (0.47–1.62)	0.674
CT vs CC+TT Codominant model	1.01 (0.71–1.45)	0.916	0.87 (0.56–1.36)	0.545
**rs7079**				
GG vs GA+AA Dominant model	1.32 (0.74–2.37)	0.347	1.04 (0.52–2.06)	0.912
AA vs GA+GG Recessive model	1.01 (0.49–2.10)	0.974	1.02 (0.44–2.40)	0.953
GA vs GG+AA Codominant model	0.50 (0.19–1.27)	0.143	0.87 (0.30−2.56)	0.805

**Abbreviations**: aOR, Adjusted Odd’s Ratio (adjusted for age, and BMI); CI, Confidence Intervals.

### Multivariate Analysis

Multivariate logistic regression was used to collectively analyse all the three polymorphisms (Table 5). As interpreted from the unadjusted odds ratio, there was no significant association (p > 0.05) between any of the polymorphisms with risk of developing hypertension. The results were the same, after adjusting for age, and waist circumference.

**Table 5:** Multivariate Logistic Regression Model of Various SNPs (*AGT* Gene) Associated with Hypertension

	Unadjusted OR (95% CI)		Unadjusted p	aOR (95% CI)	Adjusted p
**rs2004776**					
CT	1.00 (0.62–1.62)		0.985	0.88 (0.49–1.56)	0.663
TT	1.25 (0.75–2.09)		0.870	1.10 (0.59–2.04)	0.748
**rs3789678**					
CT	0.98 (0.66–1.44)		0.925	0.82 (0.52–1.31)	0.426
TT	0.87 (0.51–1.49)		0.617	0.92 (0.49–1.75)	0.821
**rs7079**					
GA	0.49 (0.19–1.26)		0.143	0.87 (0.29–2.59)	0.809
AA	0.98 (0.47–2.03)		0.961	0.93 (0.39–2.20)	0.875

**Abbreviations**: aOR, Adjusted Odds Ratio (adjusted for age, and waist circumference); CI, Confidence Intervals.

## Discussion

Hypertension is a major public health problem, particularly in Africa, contributing significantly to the increasing number of premature deaths in this region.^49^ Due to the complex genetic diversity, and inheritance patterns in Africa, genetic studies in these populations could reveal vital information about the influence of genes on human health and disease susceptibility.^50^ Many studies have investigated the association of *AGT* and SNPs with hypertension in various populations; the contradictory outcomes of these studies necessitate the replication of SNP association studies in larger groups from diverse populations around the world. To our knowledge,this is the first study to investigate the association of *AGT* polymorphisms (rs2004776, rs3789678, rs5051 and rs7079) with hypertension in a study population of isiXhosa-speaking individuals of the Eastern Cape Province, South Africa.

Consistent with other studies,^12^,^18^,^51^ the results of the present study indicate that advanced age is a risk factor in the development of hypertension. The mean age of hypertensive individuals was significantly higher than normotensive individuals. It has been postulated that increased vascular resistance may contribute to higher prevalence of hypertension among older adults.^52^,^53^

In line with previous studies, findings from the current study strongly indicate that obesity could be a risk factor in the development of hypertension in the studied population of the Eastern Cape. The present study observed significantly higher BMI in the hypertensives as compared to the normotensive controls. Previous research has found that having a higher BMI is a major risk factor for hypertension and that there is a linear association between BMI and hypertension.^18^,^54^,^55^ Obesity and visceral adiposity have also been shown to be positively linked with the RAAS, which regulates blood pressure.^56–59^ The mean waist circumference observed in the current study was significantly elevated in the hypertensive groups compared to the normotensive control group. According to the new cut-off values^60^ (≥95.25cm and ≥89.45cm for males and females, respectively), the waist circumference of the hypertensive group surpass the cut-off values; therefore, they are at greater risk of developing metabolic syndrome and hypertension.^61^

The mean hip circumference observed in the current study was significantly higher in the pooled hypertensive group compared to the normotensive controls. Although hip circumference is not directly linked with hypertension risk, it is used to calculate the waist–hip ratio, which is suggested to be a more predictive tool for the developing hypertension than waist and hip circumferences alone.^46^

The present study observed significantly higher fasting blood glucose levels in the hypertensive group than the normotensive control group. Another study found that diabetic individuals have nearly twice the risk of developing hypertension compared to non-diabetic individuals.^12^ Due to their overlapping metabolic pathways and risk factors, it is well known that there is significant commonality between hypertension and diabetes.^62^,^63^ Furthermore, a high BMI (overweight/obesity) is frequently linked to insulin resistance, which has substantial consequences for the development of type 2 diabetes mellitus.^64^,^65^ Obesity and type 2 diabetes mellitus are associated with an increased risk of death from cardiovascular illnesses.^11^,^66–68^

In the current study, genotypic, dominant, and recessive models showed no significant association between the various *AGT* SNPs (rs2004776, rs3789678, rs5051 and rs7079) and risk of developing hypertension in this study population.

Previous studies investigating the association between *AGT* polymorphisms and hypertension are limited and have reported conflicting results (Table 6).

**Table 6:** Association of AGT Polymorphisms and Hypertension in Various Populations

SNP rs Number	Nucleotide Position	Nucleotide Substitution	Cases	Controls	Population	Association	Country	References
rs2004776	Chromosome 1:230,712,956	Ancestral C C>T	782	2099	East African population	Yes	Uganda	[17]
			215	230	Indian Population	Yes	India	[69]
rs3789678	Chromosome 1:230,713,736	Ancestral C C>T	905	905	Han Chinese population	Yes	China	[70]
			202	222	Indian population	Yes	Delhi	[32]
rs7079	Chromosome 1:230,702,585	Ancestral G G>A	3521	1596	Native Saudi individuals	Yes	Suadi Arabia	[71]
rs5051	Chromosome 1:230,714,126	Ancestral T T>C	652	780	Northern Han Chinese population	Yes	China	[72]
			2203	2354	White	Yes	US	[73]
					African American	No	Suadi	
			3521	1596	Native Saudi individuals	Yes	Arabia	[71]

Consistent with our results, a study^74^ reported no association of rs2004776 with the development of hypertension in a Chinese Han population, with a similar sample size as the current study. However, in that study authors had investigated the pregnancy-induced hypertension, thus the conclusions drawn may not directly applicable to the current study. In contrast, other studies performed in African,^17^ North American^75^ and Indian^69^ populations, respectively, reported that the TT genotype (rs2004776) increased susceptibility to hypertension. However, the frequency of the minor allele found in the current study (T-allele) of rs2004776 (53%) is in line with that reported the African population (46%)^17^ but significantly higher than the frequencies observed in two Asian populations (10–30%)^69^,^74^ and a North American population (22–24%).^75^

The *AGT* rs3789678 polymorphism is not associated with hypertension in our study, and it is inconsistent with the findings reported in an Indian population.^32^ Authors identified a protective effect of this SNP in susceptibility risk of developing hypertension in an Indian population. However, three studies performed in Chinese Han populations have reported an association of rs3789678 polymorphism with an increased risk of developing hypertension.^70^,^74^,^76^ The minor allele (T-allele) frequency of rs3789678 reported in the present study is higher (37%) compared to the frequencies observed in the four Asian populations (2–14%).^32^,^70^,^74^,^77^

There was no association of rs7079 polymorphism and risk of developing hypertension in our study population. These results are contrary to the results reported by Mopidevi et al, 2013,^78^ where the rs7079 SNP showed an association with elevated BP. The C to A substitution increased circulating AGT levels by altering microRNA binding to the 3’-UTR of AGT.^78^,^79^ Micro RNAs are small noncoding RNAs of 20–22 nucleotides and have been demonstrated to regulate gene expression and various key cellular activities.^80^,^81^ A study^82^ showed that the nucleotide sequence of *AGT* gene with C allele has perfect base pairing with two micro RNAs (miR-584 and miR-31), resulting in the posttranscriptional modification of *AGT* gene and downregulating its expression, however, the presence of A allele in *AGT* gene sequence leads to destabilization of the base pairing with micro RNA and lead to increased *AGT* expression. Their results confirm that rs7079 is a functional SNP, this is consistent with the previous findings which illustrated that rs7079 is associated with non-alcoholic steatohepatitis,^83^ body fat distribution^84^ and coronary artery disease.^71^ Variants of rs7079 have also found to influence BP responses to anti-hypertensive treatments, such as benazepril in a Chinese cohort.^85^ Current literature does not provide much information on the role of this SNP in hypertension. The minor allele frequency in the current study is 9% which is like the frequency observed in the African population, however, significantly lower than the minor frequencies reported (ENSEMBL) in American (19%), European (33%) South Asian populations (29%).

The *AGT* rs5051 did not demonstrate any association with hypertension in the present study population. Similar findings were shown in a Pacific Island study, although the presence of T allele was associated with probability of having higher SBP compared to C allele carriers^86^ However, in a Saudi study,^71^ and in China,^72^ an association was reported between rs5051 and HTN. Another study also reported a significant association between the G allele of rs5051 and treatment-resistant hypertension (TRH) among Caucasians (GenHat cohorts), however, African Americans^73^ did not report any association. The minor allele frequency is 7% in the current study which is almost the same as reported in African population (8%; ENSEMBL) but significantly lower than the frequencies observed in American population (35%), European population (59%) and South Asian populations (36%).

The conflicting results of these variants in different populations possibly highlight the genetic variability in the practical relevance of these findings globally. Several factors could be responsible for these discrepancies. One of the most likely explanations is the probability of the variation in the minor allele frequencies among different populations studied.

There are some limitations in this study that can be improved in future research work. The current study had a small sample size; a larger sample size would increase the reliability and validity of results. This study only investigated the association of four SNPs of *AGT* gene. More AGT SNPs should be evaluated to explore the functional consequences of each variant and their contribution to the disease risk.

## Conclusions

In conclusion, the genotype distributions of *AGT* polymorphisms do not influence the risk of developing hypertension in the current study population of isiXhosa-speaking South Africans, and further analysis is needed on a larger sample size. Additionally, more research on study populations from other regions of South Africa is warranted to corroborate the current findings. Other polymorphisms in the coding and promoter regions of these candidate genes should also be evaluated in the context of the complex ancestral heritage of the South African population to determine their functional importance and impact on hypertension. More detailed investigations of these variants may aid in our understanding of genetic susceptibility factors in diverse populations, allowing for better prevention strategies and therapies.
